# The effect of sorghum fractions on apparent total tract digestibility and antioxidant capacity by dogs

**DOI:** 10.1371/journal.pone.0206090

**Published:** 2018-10-26

**Authors:** Isabella Corsato Alvarenga, Charles Gregory Aldrich

**Affiliations:** Department of Grain Science and Industry, Kansas State University, Manhattan, Kansas, United States of America; University of Illinois, UNITED STATES

## Abstract

Sorghum is an abundant starch source that has many potential health benefits. Some pet food companies have adopted whole sorghum in their formulations, however sorghum flour and (or) its phenolic rich seed coat might provide added benefit to companion animal diets. The objective of this experiment was to evaluate diets utilizing sorghum flour (FLD), and sorghum mill feed (MFD) relative to whole sorghum (WSD), and conventional grains (rice, corn and wheat; CON) in a typical dog diet. Adult (1–3 yr) Beagle dogs (n = 12; 10.6 kg ± 1.4) were randomly assigned to individual pens with *ad libitum* access to water. Dogs were fed twice daily and adapted to diet (9 d), and then total feces were collected for 5 d over 4 periods in a 4x4 replicated Latin square design. Fecal output for determination of digestibility was estimated using Cr_2_O_3_ as a marker. Number of defecations were quantified, and feces were scored. Approximately 3 mL of blood from each dog was collected at the end of each period and stored at -80° until further analyses. Means were separated using multivariate analysis of variance (MANOVA). Intake did not differ among treatments (average 187 g/d), but dogs fed the MFD excreted a larger (P<0.05) amount of feces, had more defecations per day, and lower (P<0.05) overall nutrient digestibility compared to the other treatments. The FLD had the highest (P<0.05) dry matter (DM), organic matter (OM), crude protein (CP) and gross energy (GE) digestibility, suggesting a possible application in “easy-to-digest” pet foods. Dogs fed the MFD had the highest (*P* < 0.05) plasma oxygen radical absorbance capacity (ORAC) value, but plasma ferulic and p-coumaric acids did not differ among treatments. Sorghum fractions have potential application in pet food; wherein, a bran rich fraction may promote antioxidant capacity, and flour increased digestibility.

## Introduction

The US pet food market was estimated to be USD 24.60 billion in 2016 with expectation to reach USD 30.01 billion in 2022 [1]. As pet ownership trends shift from mere possession to pet parenthood, food trends have shifted to selections that promote ingredients for animal health [2]. High volume industrial pet food production requires sustainable yet novel ingredient sources to support this demand.

Sorghum grain has some unique attributes that make it an interesting ingredient that merits exploration in pet foods. Some pet food companies use whole sorghum in their diets, but few to none use sorghum fractions obtained from dry milling. Sorghum bran (obtained from sorghum pericarp) was found to have a low *in vitro* glycemic index [3] and to contain phenolics in the pericarp that may improve physiological antioxidant capacity [4]. Despite these benefits, there is an off-setting challenge due to condensed tannins (also known as proanthocyanidins) within the pericarp of some sorghums which can precipitate proteins, inhibit digestive enzymes, and chelate trace minerals [5]. Conversely, as bran is removed a clean flour is produced, which may yield an improved functional starch for processing and (or) increase diet digestibility. Moreover, sorghum bran is usually discarded or fed to cattle as a result of sorghum milling during the production of flour for human consumption, and its deliberate capture and utilization would promote food sustainability. Each isolated and concentrated component, resulting from traditional grain milling techniques, may add value to sorghum and provide a novel ingredient option for use in pet foods. Yet, there is very little information available regarding composition and utilization of these potential ingredients in pet foods. Therefore, the objectives were to determine the effect of diets formulated with sorghum fractions on acceptability, utilization, and antioxidant capacity in dogs.

## Materials and methods

All animal testing was approved by the Kansas State University Institutional Animal Care and Use committee (IACUC) under protocol #3568 prior to the conduct of the study.

### Dietary treatments

Four nutritionally complete and balanced dietary treatments (Tables 1 and 2) were formulated to have similar nutrient composition. Details regarding sorghum preparation and extrusion processing have been described by Alvarenga et al. (2018). The cereal portion of the diets consisted of 1:1:1 rice, wheat and corn in the control (CON), and whole sorghum, sorghum flour and sorghum mill-feed enriched with bran and some endosperm in the WSD, FLD and MFD diets, respectively (Table 1). Chromium sesquioxide (Cr_2_O_3_; 0.25%) was included in each diet as an external marker to estimate fecal output for calculation of apparent total tract nutrient digestibility (ATTD).

**Table 1:** Experimental diets used to feed dogs: Control (CON), whole sorghum (WSD), sorghum flour (FLD) and sorghum mill-feed (MFD).

Ingredients, %	CON	WSD	FLD	MFD
Brewers rice	21.2	-	-	-
Corn	21.2	-	-	-
Wheat	21.2	-	-	-
Whole sorghum	-	64.7	-	-
Sorghum flour	-	-	63.11	-
Sorghum mill-feed	-	-	-	67.6
Chicken by-product meal	20.9	20.0	20.0	20.0
Chicken fat*	5.34	5.52	6.69	3.29
Beet Pulp	4.00	4.00	4.00	4.00
Corn gluten meal	2.35	2.35	2.35	2.35
Calcium carbonate	0.75	0.35	0.26	0.67
Potassium chloride	0.49	0.52	0.65	0.19
Salt	0.46	0.45	0.47	0.43
Dicalcium phosphate	0.87	0.95	1.27	0.24
Choline chloride	0.20	0.20	0.20	0.20
Vitamin premix^a^	0.15	0.15	0.15	0.15
Trace mineral premix^b^	0.10	0.10	0.10	0.10
Natural antioxidant	0.07	0.07	0.07	0.08
Chromium sesquioxide	0.25	0.25	0.25	0.25
Titanium dioxide	0.40	0.40	0.40	0.40

^a^Vitamin premix: calcium carbonate, vitamin E supplement, niacin supplement, calcium pantothenate, vitamin A supplement, thiamine mononitrate, pyridoxine hydrochloride, riboflavin supplement, vitamin D3 supplement, biotin, vitamin B12 supplement, and folic acid.

^b^Trace mineral premix: calcium carbonate, zinc sulfate, ferrous sulfate, copper sulfate, manganous oxide, sodium selenite, and calcium iodate.

*Applied topically following extrusion and drying.

**Table 2:** Nutrient analysis of experimental diets control (CON), whole sorghum (WSD), sorghum flour (FLD) and sorghum mill-feed (MFD) fed to dogs.

Nutrient	CON	WSD	FLD	MFD
Dry matter, %	93.9	93.6	94.8	93.8
Organic matter, %	92.7	93.5	93.4	93.1
Crude protein, %	21.5	21.4	21.2	23.8
Crude fat, %	12.10	10.7	10.25	9.48
TDF, %	4.73	7.57	4.33	16.39
Crude fiber, %	0.675	1.330	0.725	2.710
Total starch, %	46.9	45.6	50.0	35.3
Starch gelatinized of total, %	85.2	86.1	96.3	93.5
NFE (calculated), %	52.4	53.6	55.9	51.2
Ash, %	7.24	6.52	6.59	6.86

NFE = nitrogen free extract; TDF = total dietary fiber.

### Dog feeding study

Adult (1–3 yr) intact Beagle dogs (8 males and 4 female), sourced from Marshall BioResources (North Rose, NY, U.S.A.), with an average weight of 10.6 kg ±1.42, were individually housed. Cages (1.83 m x 1.20 m) were equipped with an acrylic-mesh floor over a pan to separate feces from urine. The study was conducted as a replicated Latin square design [6]. On each period dogs were fed experimental diets for 9 d adaptation and 5 d fecal collection.

**Fig 1:**
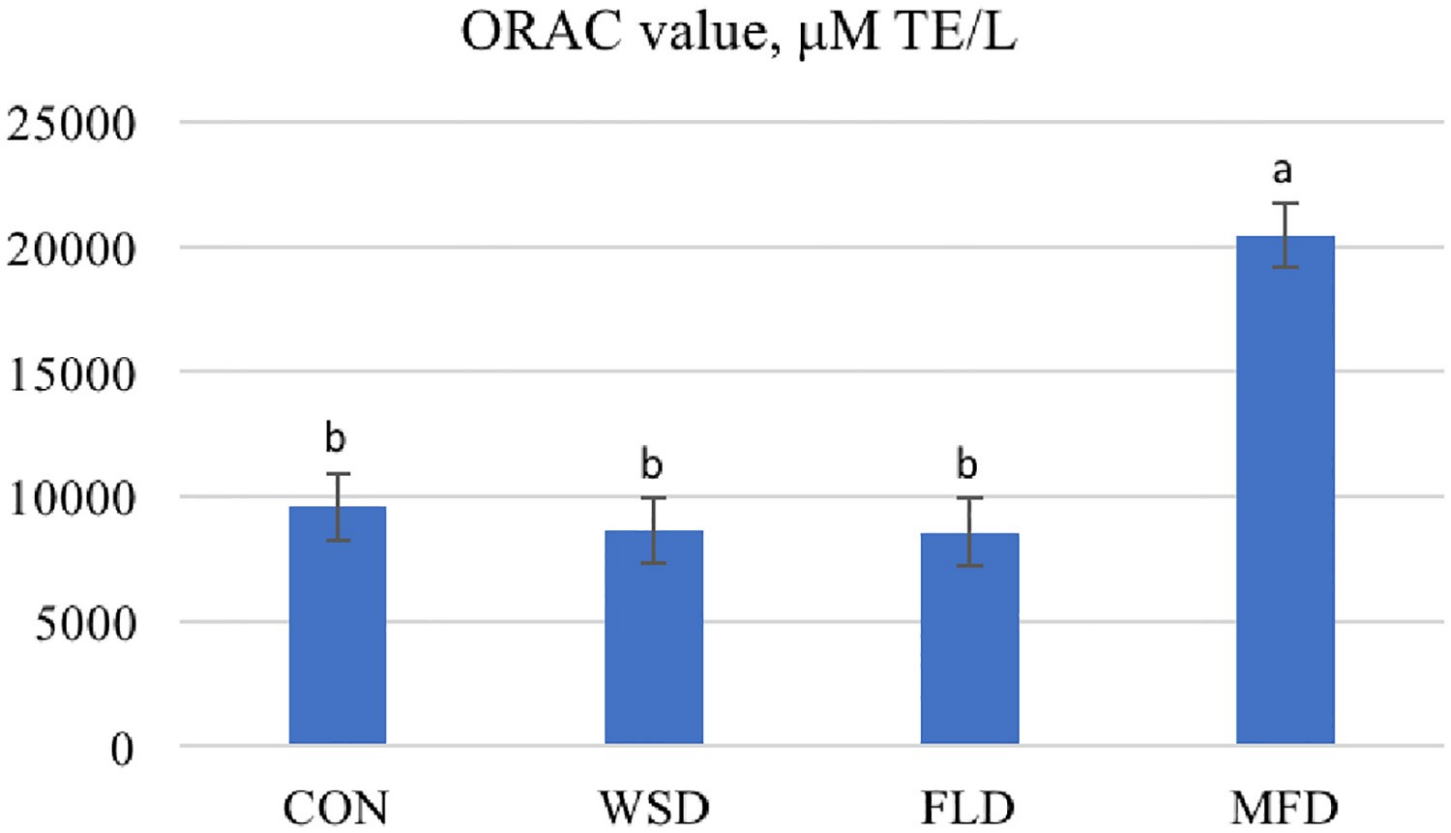
Five-point fecal scoring chart used to score feces of dog fed control (CON), whole sorghum (WSD), sorghum flour (FLD) and sorghum mill-feed (MFD) diets.

Dogs were fed twice daily (08:00 and 17:00) an amount of food estimated to maintain body weight, based on estimates of energy requirements calculated from NRC [7] in which the metabolizable energy (ME) was determined as (kcal/day) = 130 x BW^0.75^. The dietary energy values were estimated from modified Atwater [8] values [ME (kcal/kg) = 3.5 x NFE + 8.5 x (Crude Fat) + 3.5 x (Crude Protein)]. Dogs had free access to water at all times and were housed in a climate-controlled building at 22–23°C, with a 16h light/8h dark cycle. During the collection period unconsumed food on offer was collected and weighed. All feces excreted during the 120 h of each collection period were scored for consistency, placed in plastic bags, and stored at -15°C until further analysis. The number of defecations were recorded each day and stools were scored using a 5-point scale in half point increments wherein: 1 = watery-liquid that can be poured; 2 = soft, unformed stool that assumes shape of container; 3 = softer stool that retains shape; 4 = hard, formed stool (ideal); 5 = very hard, dry pellets (Fig 1; Table 3). Approximately 3 mL of blood from each dog was collected into test tubes with ethylenediaminetetraacetic acid (EDTA) at the last day of each period and placed in ice. Within an hour after blood collection, plasma was separated by centrifugation and stored at -80° until phenolic acids and antioxidant capacity analyses.

**Table 3:** Food intake, feces collected (on dry matter basis) per day, number of defecations per day, fecal scores and apparent total tract digestibility (*ATTD*) of dogs fed control (CON), whole sorghum (WSD), flour (FLD) and mill-feed (MFD) diets (N = 12).

Item	CON	WSD	FLD	MFD	SEM	*P*
Food intake, g/d	185	186	181	195	6.5	0.4818
Wet feces, g/d	75.2^c^	95.6^b^	60.6^c^	159.7^a^	5.03	<.0001
Dry feces, g/d	42.0^c^	55.7^b^	32.6^c^	95.4^a^	3.24	<.0001
Defecations per day	2.18^b^	2.38^b^	2.10^b^	3.02^a^	0.098	<.0001
Fecal score (1–5)	3.60^b^	3.68^a^^b^	3.78^a^^b^	3.92^a^	0.068	0.0007
*ATTD*, *%*						
Dry Matter	83.0^b^	81.1^c^	86.0^a^	65.9^d^	0.44	<.0001
Organic Matter	88.0^b^	85.8^b^	90.7^a^	70.6^c^	0.34	<.0001
Gross Energy	87.2^b^	85.4^b^	90.3^a^	70.2^c^	0.68	<.0001
Crude Protein	77.5^b^	76.3^b^	81.8^a^	67.2^c^	0.73	<.0001
Crude Fat	91.5^a^	88.4^b^	91.4^a^	77.9^c^	0.37	<.0001
NFE (calculated)	94.2^b^	91.6^c^	96.3^a^	77.0^d^	0.35	<.0001
TDF	13.7^b^	27.7^a^	30.2^a^	10.3^b^	1.94	<.0001

^abc^ Means within a row that lack a common superscript differ (*P* < 0.05).

NFE = nitrogen free extract; TDF = total dietary fiber.

### Digestibility estimation

Collected feces of each dog from each period were dried separately on an aluminum pan in the oven (Cat. #52755–20, Matheson Scientific, Morris Plains, NJ) at 55°C until weight decline ceased (24h-48h), and then ground to 1 mm in a laboratory fixed blade impact mill (Retsch, type ZM200, Haan, Germany). Chromium concentration was measured in feces and food by atomic absorption spectrophotometry [9]. Apparent total tract digestibility (ATTD) was calculated according to Eq 1:

$$Nutrient\;digestibility\;=\;\frac{[1-(\%\;Cr\;in\;food\;\times \%\;nutrient\;in\;feces)]\times 100}{\left(\%\;Cr\;in\;feces\;\times \%\;nutrient\;in\;food\right)}$$ (1)

In Eq 1, “nutrient” was used to represent dry matter, organic matter, gross energy, crude protein, crude fat, NFE and TDF.

### Nutrient analysis

Moisture and dry matter (AOAC 930.15), organic matter and ash (AOAC 942.05), crude protein (AOAC 990.03), crude fat by acid hydrolysis (AOAC 954.02), and crude fiber (AOCS Ba 6a-05) were measured in fecal and food samples at a commercial analytical laboratory (Midwest Laboratories, Omaha, NE, U.S.A.). Total dietary fiber (TDF; AOAC 985.29) was measured on fecal and food samples using a kit (TDF-100A; Sigma-Aldrich; Saint Louis, MO, U.S.A.). Total starch (AACC 76–11; modified) and starch gelatinized (AACC 76–11) were only measured in the diets (Midwest Laboratories, Omaha, NE, U.S.A.).

### Phenolic acids analysis

Food and plasma sample preparation and analysis for phenolic acids analysis followed the protocol described by Titgemeyer et al. [10]. However, after the extraction of phenolics from plasma samples, methanol was evaporated with N_2_ until dried, and then diluted with 0.3 mL 50% methanol. This additional step was required to concentrate phenolic acids in plasma to measurable levels. The HPLC separation was conducted with a 250 x 4.6 mm Spherisorb S5 ODS2 C18 reversed-phase column. Gradient elution was performed with a mobile phase of n-butanol: acetic acid (350: 8.5: 1) mixed by volume (solvent A) and a mobile phase of 100% methanol (solvent B): 0 min, 0% solvent B; 8–15 min, 0–25% solvent B; 15–20 min, 25% solvent B; 20–30 min, 0% solvent B. Chromatography was performed at 41°C with a flow rate of 1.85 mL/min and injection volume of 20 μL. Phenolic acids were detected at a wavelength of 305 nm (UV).

### Antioxidant capacity

Dog plasma was stored frozen (-80 °C) for 10 months before antioxidant capacity was measured using the oxygen radical absorbance capacity (ORAC) method (Cell Biolabs Inc.; CA, U.S.A.). Plasma sample preparation and kinetic fluorescence procedures were performed according to protocol. Fluorescence was recorded every 1 min for 60 min at excitation and emission wavelengths of 480 and 520 nm, respectively, on a plate reader (Gen5; Biotek Instruments, Inc.Winooski; VT, U.S.A.). Results were calculated according to the standard curve (Gen5 Microplate Data Collection and Analysis Software).

### Statistical analysis

The experiment was conducted as a 4 x 4 replicated Latin Square design. Each of the 12 experimental units (dogs) were randomized to treatment and replicated using the spreadsheet by Kim and Stein [6]. The model statement contained dog, period and diet as fixed variables. Means were separated by multivariate analysis of variance (MANOVA) with the aid of a statistical software (SAS; v 9.4) and grouped using Bonferroni correction for food intake, wet and dry feces, defecations per day, fecal scores, ATTD, and antioxidant potential. Phenolic acids means were corrected by the Tukey method. Means were considered different at a *P* < 0.05.

## Results

### Dietary treatments

The 4 dietary treatments were formulated to a similar level of protein, fat, minerals and vitamins, with different fiber contents due to their individual cereal composition (Table 1). The final moisture among diets of 5.92% met the production target (< 7.5%; Table 2). Slight deviations in crude protein and crude fat from target were noted. Specifically, the crude protein level was slightly higher for MFD than expected from previous milling tests [11]. For crude fat the CON was 2.62% points greater than the MFD with WSD and FLD intermediate. The total dietary fiber (TDF) matched the higher expected concentration in the MFD relative to other diets. Crude fiber of diets followed the same ranking as TDF, but were lower because the method does not account for all soluble fibers. The starch was greater in CON, WSD and FLD (average 47.5%) and lower in MFD (35.3%). Of the starch present in the diets, gelatinization for FLD and MFD was greater than CON and WSD.

### Dog feeding study

Food allowance was intended to maintain animal body weight, but a slight weight loss was observed. Dogs lost an average of 1.95%, 1.37%, 2.48% and 3.35% body weight when fed the CON, WSD, FLD and MFD, respectively. No adverse events occurred during the feeding trial and all dogs completed the study. The daily amounts were consumed readily by dogs and did not differ among treatments (average 187 g/d). Total wet feces collected were greatest (*P* < 0.05) when dogs were fed MFD, intermediate when dogs were fed WSD and lowest for those fed CON and FLD (160 vs 95.6 and average 67.9 g/day, respectively; Table 3). The dried fecal weights followed the same rank order. The number of defecations per day were greater (P<0.05) for dogs were fed the MFD than dogs fed CON, WSD or FLD (3.02 vs average 2.22 times per day, respectively). While there were some subtle differences in stool scores among the treatments, all were considered acceptable with an average score > 3.5. Further, all treatments had at least one fecal score of 2 and one fecal score of 5 (range 2–5), with a median of 4.

### Apparent total tract digestibility

Apparent total tract digestibility of DM, OM, gross energy, and protein was greatest (*P* < 0.05) for FLD and lowest for MFD (Table 3). The CON and WSD were intermediate and did not differ from each other, except for DM ATTD where CON was higher (*P* < 0.05) than WSD. There was a slight deviation in the rank order for crude fat ATTD in that the WSD was lower (*P* < 0.05) than CON or FLD, but greater than MFD. As expected, the total dietary fiber ATTD was low across all treatments. Among all nutrients, MFD fed dogs had lower ATTD, while FLD tended to improve overall nutrient disappearance.

### Antioxidant capacity

**Fig 2:**
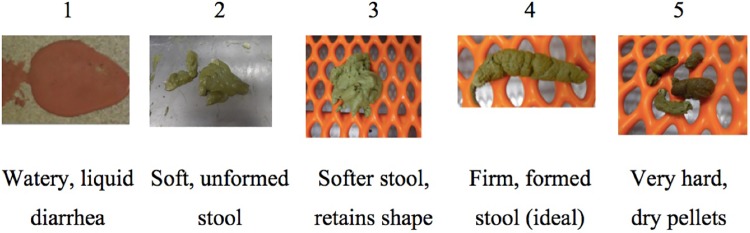
Oxygen radical absorbance capacity (ORAC) of plasma collected from dogs at the end of each period fed control (CON), whole sorghum (WSD), sorghum flour (FLD) and sorghum mill-feed (MFD) diets (N = 12; *P* < .0001). TE = Trolox equivalent. ^ab^Means within a row that lack a common superscript differ (*P* < 0.05).

Results from the ORAC analysis indicated that dogs fed the MFD had an antioxidant capacity over 2-fold greater (*P* < 0.05) than each of the other treatments CON, FLD and WSD (20,482 vs average 8,923 μM TE/L; Fig 2). In regard to the phenolic composition initial assessment of cereal sources, the whole corn had the greatest p-coumaric (PCA) and ferulic (FA) acid concentration relative to sorghum mill-feed (410 and 2711 ppm vs 372 and 1311 ppm, respectively; Table 4). Rice, sorghum and wheat flour had the lowest FA. Wheat flour and whole sorghum FA concentration were intermediate (average 525 ppm). For the diets, PCA concentration in MFD was more than 2 times the concentration of CON, WSD or FLD. Ferulic acid was also more concentrated in MFD than in WSD and FLD, and CON was intermediate. Although diets were measured with different concentrations of FA and PCA, plasma FA and PCA of dogs fed experimental diets did not differ among treatments (averages 0.143 and 0.095 ppm, respectively; Table 4).

**Table 4:** Ferulic and p-coumaric acids concentrations of starch ingredients, diets, and plasma from dogs fed control (CON), whole sorghum (WSD), sorghum flour (FLD) and sorghum mill-feed (MFD) diets.

	Ferulic acid, ppm	P-coumaric acid, ppm
*Ingredients*^1^		
Whole sorghum	633	110.7
Sorghum flour	397	45.0
Sorghum mill-feed	1311	372.2
Whole corn	2711	410.4
Wheat flour	416	36.3
Brewers rice	132	11.2
*Diets*^1^		
CON	1025	155
WSD	742	133
FLD	574	100
MFD	1425	314
*Plasma*		
CON, *N* = 11	0.100	0.014
WSD, *N* = 10	0.078	0.011
FLD, *N* = 12	0.158	0.013
MFD, *N* = 11	0.084	0.023
*MSE*	*0*.*0111*	*0*.*0004*
*P*	*0*.*4285*	*0*.*5062*

^1^Phenolic acids concentration in ingredients and diets are expressed as means of two analytical replicates.

## Discussion

In this study, food intake was restricted to meet animal needs for maintenance of body weight. Because of this, dogs consumed their diets readily and no differences in intake were observed relative to treatment. Kore et al. [12] and Teixeira [13] also reported that diets containing significant amount of sorghum (> 50%) did not influence intake by dogs. The greater amount of feces excreted daily, and more defecations per day by dogs fed the MFD did not correspond with lower stool scores. Instead, dogs fed the MFD had the highest stool scores along with the greatest wet fecal weights. This suggests that the fiber present in sorghum mill feed trapped water inside the fecal mass. The same phenomenon was observed in a study in which dogs fed diets containing sorghum produced larger quantities of feces without affecting fecal scores [13]. Humans fed sorghum bran also had increased stool weight, number of defecations and decreased intestinal transit time [14]. These results were likely observed because sorghum bran is richer in insoluble than soluble fiber [15], which leads to increased stool bulk and defecation frequency. When comparing whole sorghum with other starch ingredients fed to dogs, other studies [16, 12] also reported no effect on fecal scores.

In the present study, the digestibility of nutrients by dogs fed the MFD were much lower than the other treatments. For DM, OM and energy, the MFD treatment led to an ATTD 20 percentage units lower than the FLD treatment. A study evaluating the effects of fiber on ATTD digestibility by Beagle dogs reported a decrease of 2.2% in the DM ATTD for every percent of added cellulose [17]. Cellulose is an insoluble fiber similar to that found in sorghum bran, so the low digestibility of the MFD was likely due to its high fiber content. Dogs fed WSD resulted in similar ATTD to what has been reported in the literature (OM ATTD = 85.8%; DM ATTD = 81.1%). For example, Kore et al. [12] reported that dogs fed a diet containing 70% whole sorghum had a DM and OM ATTD of 83.1 and 85.1%, respectively. This is similar in magnitude to contemporary animals fed a rice or corn-based diet. Carciofi et al. [18] also reported that the DM and OM ATTD of dogs fed a diet containing nearly 60% sorghum were close to 80%, and similar to results from dogs fed a corn based diet. The FLD had the highest DM and OM digestibility, when compared to the digestibility of a cassava flour based diet [18].

Dogs fed WSD had a protein digestibility similar to CON, but they were both lower than what others have observed. For instance, Kore et al. [12] reported a protein digestibility for a sorghum-based diet to be over 90% and similar to diets containing rice and maize as the only starch source. The CP ATTD of dogs fed a sorghum-based diet was reported to be 85.0% and similar to a cassava flour, corn and pea-based diets [18]. Murray et al. [19] reported that crude protein disappearance of a sorghum containing diet fed to dogs had similar digestibility to diets containing barley, potato, rice, and (or) wheat. Dogs fed the MFD had low CP digestibility, which followed the same trend as the other nutrients. The high TDF (16.4%) present in the MFD led to lower overall nutrient absorption, which agrees with what has been reported about other fiber sources [20, 21].

Further, the TDF ATTD of dogs fed the MFD was the only nutrient digestibility similar to the CON treatment. All values were low for ATTD of TDF. This is consistent with other research [18, 22]. By the very nature of fiber being an indigestible component and sorghum bringing insoluble fiber to the formula, these results were not unexpected. Though it would be interesting to evaluate the fermentability of the mill feed fiber structure by colonic microorganisms.

The present work also explored health effects provided by phenolic acids, which behave as antioxidants. This is supposedly due to the reactivity of the aromatic ring structure of the molecules that are believed to be radical scavenging via hydrogen atom donation [23]. Ferulic (FA) and p-coumaric (PCA) are the most abundant phenolic acids in red sorghum [24], so these were measured in the cereals, diets and dog plasma. Sorghum mill-feed was expected to contain the highest PCA and FA, but was second to corn. Adom and Liu [25] also measured high FA in corn (1,740 ppm). Of course, when the remaining dietary ingredients were incorporated into the diets, the high phenolic acid concentration in corn was much more diluted by rice and wheat in the CON. Thus, the MFD resulted in the highest PCA and FA. Despite this, phenolics were not elevated in plasma and yet dogs fed the MFD had twice as much antioxidant potential than the other treatments when assessed by the ORAC method. The ORAC method was chosen for this study because it is the only standardized *in vitro* method that employs biologically relevant free radicals [26]. A study has reported that the ORAC method is reproducible and sensitive to be used in plasma of many animal species, including dogs [27]. Another study found that dogs fed some diets supplemented with antioxidants had a significant increase in plasma ORAC value compared to a control diet [28]. In our study the elevated ORAC values reported in Fig 1 may be due to other constituents other than phenolic acids. This may indicate that the high antioxidant potential of MFD was due to phenolic derivatives of PCA and FA not measured in this experiment, and (or) conjugated forms of phenolic acids, which are commonly found in plasma [29, 30]. Some constituents of the plasma such as vitamin E, vitamin C, thiols and uric acid may also interfere with the ORAC assay. Inferences about the true antioxidant benefit from sorghum bran might be better elaborated in an *in vitro* digestion model to fully characterize the range of antioxidant molecules.

## Conclusions

Based on this work pet food companies could consider sorghum and its various fractions as viable new ingredients in their recipes. In this study dogs who were fed the MFD produced more feces and had overall lower nutrient digestibility. While viewed from this factor alone sorghum bran rich in fiber and phenolic compounds might be considered negative. However, these features may actually be beneficial to calorie control for obese or diabetic pets, or those with special conditions benefiting from antioxidant fortification. The magnitude of reduction in digestibility does not appear to be negative for the health of dogs, but when formulating a weight loss diet the low protein digestibility in the MFD must be considered. In this situation it would be suggested that sorghum mill-feed be complemented with a highly digestible protein ingredient. Conversely, sorghum flour provided a slight improvement to digestion coefficients and might have application in “easy to digest” formula needs. The mill-feed diet had the greatest phenolic concentration, which may account for the higher antioxidant potential in dog plasma according to the ORAC method. Yet, phenolic acids in plasma did not account for this increase. This suggests that phenolic acids may be present in a different form in the blood. A future area of research should determine the optimum amount of sorghum mill-feed to incorporate in a food to benefit health while retaining overall nutrient utilization.

## Supporting information

S1 File ARRIVE guidelines checklist (fillable). (PDF)

S2 File Alvarenga sorghum digest. (XLSX)
